# Outcome of Fontan Patients After Reaching Adolescence: The Impact of Hypoplastic Left Heart Syndrome

**DOI:** 10.3390/jcm14238611

**Published:** 2025-12-04

**Authors:** Pinar Bambul Heck, Andreas Schüttler, Alfred Hager, Masamichi Ono, Jürgen Hörer, Peter Ewert, Oktay Tutarel

**Affiliations:** 1 Department of Paediatric Cardiology and Congenital Heart Disease, TUM University Hospital German Heart Center, 80636 Munich, Germany; uni.andi20@gmail.com (A.S.); hager@dhm.mhn.de (A.H.); ewert@dhm.mhn.de (P.E.); 2 Europäisches Kinderherzzentrum München, 80636 Munich, Germany; ono@dhm.mhn.de (M.O.); hoerer@dhm.mhn.de (J.H.); 3 Department of Congenital and Pediatric Heart Surgery, TUM University Hospital German Heart Center, 80636 Munich, Germany; 4 Division of Congenital and Pediatric Heart Surgery, University Hospital of Munich, Ludwig-Maximilians-Universität München, 81377 Munich, Germany; 5 Deutsches Herzzentrum der Charité, Department of Congenital Heart Disease—Pediatric Cardiology, Augustenburger Platz 1, 13353 Berlin, Germany; 6 Charité—Universitätsmedizin Berlin, Corporate Member of Freie Universität Berlin and Humboldt-Universität zu Berlin, Charitéplatz 1, 10117 Berlin, Germany

**Keywords:** Fontan, single ventricle, hypoplastic left heart

## Abstract

**Background/Objectives**: Data on the long-term outcomes of hypoplastic left heart syndrome (HLHS) patients compared to other single-ventricle patients reaching adolescence after Fontan surgery is limited. This study analyzes the outcomes of HLHS patients compared to non-HLHS patients following total cavopulmonary connection (TCPC) from the same era at a large single center. **Methods**: This study included patients aged ≥ 12 years at the last follow-up who underwent TCPC surgery between 05/2001 and 12/2009, with follow-up data available from 05/2012 to 01/2024. The primary endpoint (Fontan-specific major adverse cardiovascular events, MACEs) included all-cause death, cardiac transplantation or listing, heart failure hospitalizations, ventricular arrhythmias, third-degree AV block, or resuscitation. **Results**: A total of 130 patients were included, with 39 (30.0%) having HLHS. Among non-HLHS patients, 18 (13.8%) had a systemic right ventricle, and 73 (56.2%) had a systemic left ventricle. The mean age at the last follow-up was 18.6 ± 3.2 years, with no significant age difference between groups (*p* = 0.195). HLHS patients experienced significantly more MACEs (*p* = 0.019), had reduced ventricular function (*p* = 0.009), and exhibited higher NT-proBNP levels (*p* = 0.004) compared to non-HLHS patients. **Conclusions**: While long-term outcomes for adolescents with TCPC are generally encouraging, HLHS patients are at higher risk of adverse cardiovascular events. These findings highlight the need for targeted follow-up and interventions to improve long-term prognosis in this high-risk group.

## 1. Introduction

Fifty years after Fontan and Baudet pioneered the first surgical treatment for a congenital heart defect with single-ventricle (SV) physiology [1], significant progress has been made in both medical and surgical treatment in the ongoing care of patients with functionally SV physiology [2].

Over time, modifications of the original Fontan operation have aimed to optimize hemodynamics and reduce postoperative complications. In contemporary practice, the total cavopulmonary connection (TCPC) represents the modern modification of the Fontan procedure, providing a more efficient and widely adopted approach to single-ventricle palliation. Despite these advances, the Fontan circulation remains a “palliative” rather than curative solution, associated with risks of ventricular dysfunction, multi-organ impairment, and eventually “failing Fontan” circulation, which carries a poor prognosis [3,4]. Furthermore, protein-losing enteropathy and plastic bronchitis remain severe complications [5]. Numerous risk factors have been identified for early Fontan failure, including systolic and diastolic ventricular dysfunction, atrioventricular valve dysfunction, elevated pulmonary pressure, or lymphatic abnormalities [6,7,8]. The influence of ventricular morphology (right- versus left-dominant ventricles) on Fontan outcomes has been the focus of considerable research [9,10]. Several studies have demonstrated poorer early postoperative outcomes in patients with right ventricular morphology, while long-term results remain controversial [11,12]. This is particularly relevant for patients with hypoplastic left heart syndrome (HLHS), who possess a single systemic right ventricle and represent one of the most complex subgroups undergoing Fontan palliation. Population-based data indicate that HLHS remains one of the congenital heart defects most strongly associated with mortality, particularly during infancy [13].

The Norwood procedure, the first-stage palliation for hypoplastic left heart syndrome, carries relatively high mortality due to the complex neonatal physiology and demanding surgical reconstruction required to establish systemic and pulmonary circulation. Mortality is largely driven by the technical challenges of arch reconstruction and shunt creation, the fragile hemodynamic balance of single-ventricle physiology, and postoperative risks such as low cardiac output, shunt obstruction, and inadequate systemic perfusion [14]. Advances in surgical technique, perioperative management, and interstage monitoring have improved survival; however, data on long-term outcomes of HLHS patients who reach adolescence or adulthood remain limited. It is important to investigate whether patients with HLHS, who survive the high-risk infancy phase, particularly after Norwood procedure, experience comparable long-term outcomes to those with Fontan circulation who do not have HLHS.

The aim of this study is to assess the long-term outcomes of adolescents and adults with hypoplastic left heart syndrome in comparison with those of patients with other types of single-ventricle physiology—such as double inlet left ventricle (DILV), tricuspid atresia (TA), or double outlet right ventricle (DORV)—who underwent total cavopulmonary connection (TCPC) during the same era at a large tertiary care center.

## 2. Materials and Methods

All patients who underwent TCPC surgery between May 2001 and December 2009 with available follow-up data between May 2012 and January 2024 were included in this study. Start of follow-up was the 12th birthday of the patient. In order to reduce potential follow-up bias related to the timing of Fontan completion [15], as later completion has been associated with poorer long-term outcomes, only patients who underwent the procedure before the age of six years were included in the study. The study period was initiated in 2001, coinciding with the establishment of a structured surgical program for hypoplastic left heart syndrome at our institution, although isolated procedures had been performed since the 1990s.

This study was designed to evaluate long-term outcomes in patients who survived at least 12 years after Fontan completion. Early postoperative and interstage mortality were not included in the analysis, as these outcomes are driven by distinct perioperative factors and have been extensively studied previously. The present analysis focuses on long-term survivors to assess late morbidity and functional outcomes during adolescence and adulthood.

Patients with functionally single-ventricle physiology who did not undergo TCPC were excluded from the analysis.

Clinical data were collected retrospectively from medical records, including preoperative and perioperative characteristics, in-hospital and outpatient documentation, laboratory values, functional status, echocardiographic findings, cardiac catheterization results, occurrence of major cardiovascular events, mortality, and cause of death.

HLHS was defined by the presence of mitral stenosis or atresia, aortic stenosis or atresia, and a diminutive left ventricle. The diagnosis of Fontan-associated liver disease (FALD) was retrieved from hepatology reports documented in the patients’ medical records. All patients were evaluated by specialized hepatologists as part of the multidisciplinary Fontan follow-up program. The diagnosis was based on comprehensive hepatologic assessment, including clinical evaluation, laboratory testing (liver function and cholestatic parameters), and imaging studies (ultrasound and/or elastography), and, when indicated, liver biopsy or hepatic MRI. As the diagnosis was made by hepatology specialists according to contemporary clinical standards rather than predefined study-specific thresholds, no additional operational criteria (e.g., fixed cutoff values for GGT, AST, or elastography) were applied.

### 2.1. Laboratory Values

Data from routine in- and outpatient laboratory evaluations was analyzed. This included one of the latest laboratory workups from the routine follow-up examinations. The biochemical profile included complete blood count, liver enzymes (gamma-glutamyl transferase, GGT; alanine-aminotransferase, ALT; aspartate-aminotransferase, AST), creatinine, blood urea nitrogen (BUN), and N-terminal pro-brain natriuretic peptide (NT-proBNP).

### 2.2. Echocardiography

Quantitative echocardiographic estimates of systolic and diastolic ventricular function were not feasible due to the complex and heterogeneous anatomy in Fontan circulation, particularly in HLHS with a systemic right ventricle. Therefore, the systolic ventricle function was graded, based on visual ventricular assessment, as normal, mildly reduced, and reduced by two experienced congenital echocardiographers, each blinded to clinical data. Atrioventricular valve regurgitation was classified according to the current recommendation of the American Society of Echocardiography as none, trivial, mild, moderate, or severe [16]. All echocardiographies were performed on a VingMed System Five (GE Healthcare).

### 2.3. Endpoints

The primary endpoint, a Fontan-specific major adverse cardiovascular event (MACE), was defined as a composite of all-cause death, cardiac transplantation or listing for transplantation, hospitalizations due to cardiac decompensation (including peripheral edema, pleural effusion, dyspnea), ventricular arrhythmias, third-degree AV-Block, or resuscitation. Patients who had one of these events were censored. The secondary endpoint (extended MACE) included, in addition to the aforementioned endpoints, the occurrence of a thromboembolic event (i.e., stroke, major pulmonary embolus, or Fontan circuit thrombus with circulatory failure), further cardiac catheter interventions, cardiac surgeries, or failing Fontan circulation with protein-losing enteropathy (PLE) or plastic bronchitis. Only major cardiovascular events, surgeries, and cardiac interventions after the age of 12 years were included in the statistical analysis. Two congenital cardiologists independently adjudicated adverse events.

### 2.4. Statistical Analysis

Statistical analyses were performed with the software SPSS version 28 (IBM Corp., Armonk, NY, USA) and MedCalc version 23.01 (MedCalc Software, Mariakerke, Belgium). Continuous variables are presented as mean, standard deviation, or median (interquartile range), and categorical variables as numbers (percentage). The Mann–Whitney U test or Student’s *t*-test was used for comparison between groups for continuous variables, while the Chi-square test was used for categorical variables. Kaplan–Meier curves and log-rank tests were used to compare the occurrence of the primary endpoint. All tests were performed two-sided and, for all analyses, a *p*-value < 0.05 was considered statistically significant.

## 3. Results

In total, 131 patients met the inclusion criteria. All patients underwent TCPC with an extracardiac conduit. Patients were categorized according to ventricular dominance: 72 patients had a dominant left ventricle (55.0%), 58 patients had a dominant right ventricle (44.2%), and 1 patient had an ambiguous ventricular morphology. The latter patient was excluded from the further statistical analysis. Of the 58 patients with dominant right ventricle, 39 (67.2%) had HLHS. The patients’ characteristics and underlying cardiac anomalies are presented in Table 1. The subgroups did not differ significantly in age at surgery (*p* = 0.30) or in total follow-up time (*p* = 0.195).

### 3.1. Survival

A total of 3 out of 130 (2.3%) patients died during the follow-up period. One patient had dominant left ventricle and CAVSD, and two patients had HLHS. All patients died due to cardiac failure. The patients with HLHS had failing Fontan with severe ascites and pleural effusions.

### 3.2. Primary and Secondary Endpoints

**Figure 1:**
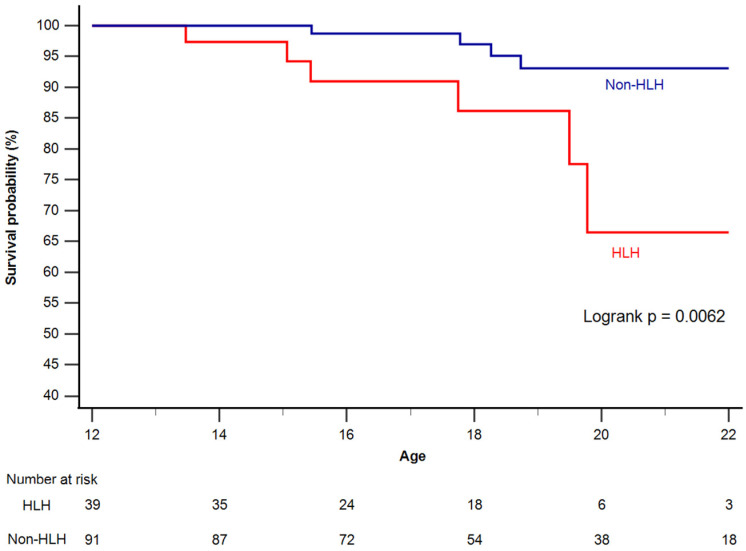
Kaplan–Meier curves for survival free from occurrence of the primary endpoint.

During a median follow-up of 18.2 years (IQR 15.7–21.4 years), the primary endpoint occurred in 11 patients (8.5% of all patients) after their 12th birthday. It occurred in six patients with HLHS (15.4%) and five patients (5.5%) with non-HLHS. Survival free from the primary endpoint according to the type of the underlying cardiac defect (HLHS vs. non HLHS) is depicted in Figure 1.

A total of 10 out of 130 patients (7.6%) had a failing Fontan circulation. Patients with HLHS tended to have a higher incidence of failing Fontan compared to non-HLHS patients, 10.3% vs. 6.7%, although the difference was not statistically significant (*p* = 0.489). Patients with HLHS had significantly higher rates of Fontan-associated liver disease, *p* = 0.017.

**Figure 2:**
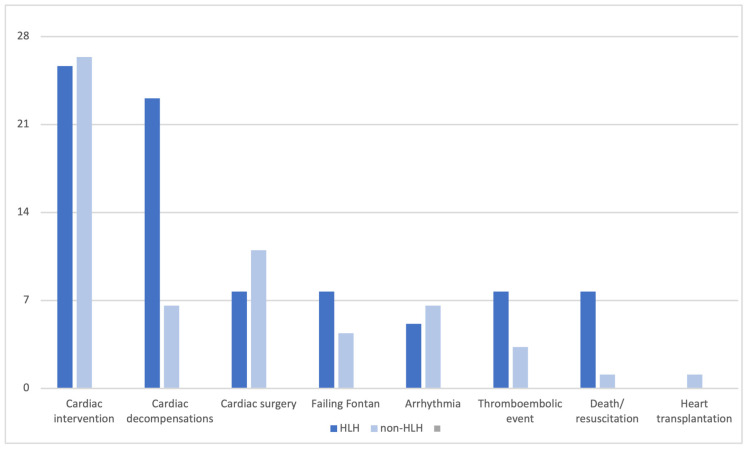
Overview of extended major adverse cardiovascular events (MACEs) in patients with HLHS and in non-HLHS.

Regarding the occurrence of the secondary endpoint, there were no significant differences between HLHS patients and patients with other underlying cardiac anomalies (*p* = 0.546). An overview of the primary and secondary endpoint occurrences in HLHS and non-HLHS patients is provided in Figure 2.

The numbers reflect the proportion of different MACEs within the total MACE count within the group of patients with HLHS and Fontan patients other than HLHS in percent.

### 3.3. Clinical Outcome

At the last follow-up, 68.3% of the study population were in New York Heart Association functional class I/II. Regarding functional class, there was no significant difference between the subgroups (*p* = 0.613).

A total of 27 cardiac interventions were performed in 91 non-HLHS Fontan patients (29.7%), while 12 interventions were performed in patients with HLHS (30.8%). Thirty-nine catheter interventions were performed at a mean time of 7.6 ± 4.1 years postoperatively. These included collateral and venovenous fistula closure in 17 patients, stent implantation or balloon angioplasty of the left pulmonary artery in 13 patients, aortic arch stenosis with ballon angioplasty or stent implantation in 5 patients, and stent implantation and/or angioplasty of the TCPC tunnel in 3 patients.

Late reoperations were performed in 15 patients, including atrioventricular valve (AVV) procedure in 3 patients (1 patient with HLHS and in 2 non-HLHS patients), aortic valve repair/replacement in 3 (1 patient with HLHS and in 2 non-HLHS patients), pulmonary artery reconstruction in 1 HLHS patient, and 6 pacemaker surgeries, all in non-HLH patients.

The systemic ventricular function and the degree of AVV regurgitation were reviewed from the last available echocardiographic report. Patients with HLHS had significantly reduced ventricular function and higher rates on AVV regurgitation (*p* = 0.009 and *p* = 0.044, respectively). NT-proBNP levels were significantly higher in patients with HLHS, *p* = 0.004. Other laboratory parameters, including BUN, creatinine, ASAT, ALAT, GGT, albumin, and serum protein, did not show any significant difference between patients with HLHS and non-HLHS.

An overview of follow-up dates is given in Table 2, and an overview of cardiac interventions is given in Table 3.

## 4. Discussion

In this retrospective single-center study, we report long-term survival and the incidence of major cardiovascular events in adolescent and adult patients undergoing single-ventricle palliation. Our results demonstrate that, over a follow-up period of up to 22 years after TCPC, patients with HLHS experienced significantly more adverse cardiovascular events compared to those Fontan patients with other underlying congenital heart defects.

As an increasing number of patients with Fontan circulation—particularly those with HLHS—reach their third decade of life, the long-term sequelae of Fontan physiology are becoming more evident. Our findings indicate that patients surviving into adolescence and adulthood after Fontan completion generally have a favorable prognosis, especially those with TCPC. These observations are consistent with previous registry data reporting encouraging survival rates in this population [17,18].

Many of these individuals maintain good functional status (NYHA I/II). Despite the anatomical and physiological complexity of their conditions, overall mortality among young adult survivors beyond the second decade remains low. However, HLHS patients demonstrated significantly reduced ventricular systolic function and higher degrees of atrioventricular valve regurgitation, reflected by elevated NT-proBNP levels in our cohort. Furthermore, HLHS patients exhibited significantly higher rates of Fontan-associated liver disease (FALD), likely due to chronically low cardiac output and elevated central venous pressure. FALD is a frequent long-term complication after Fontan surgery and may limit survival through progression to cirrhosis or the development of hepatocellular carcinoma [19].

Similarly to the findings of Wilson et al., in a multicenter study with 59 HLHS patients, our HLHS cohort demonstrated a higher prevalence of major adverse cardiovascular events (MACEs) upon reaching adolescents with a prevalence of 21% [20]. Additionally, 10% of HLHS patients in our study population had failing Fontan circulation, a condition associated with significant morbidity and mortality. This highlights a key distinction between patients with HLHS and those who underwent Fontan operation for other diagnoses.

A major strength of our study is the comparison of Fontan patients from one large single center, all operated on during the same time period by the same surgical team and following the same perioperative and postoperative care protocols. This homogeneity minimizes inter-institutional variability and potential treatment-related bias.

A notable proportion of patients required additional cardiac interventions or surgeries. In our study, nearly one-third of the patients, regardless of the underlying cardiac defect and dominant ventricle morphology, needed cardiac interventions, while 15 patients required a surgical procedure.

When analyzed by subgroup, HLHS patients experienced a higher frequency of major cardiovascular events compared to those with Fontan circulation for other diagnosis. Numerous studies have reported that right ventricular dominance significantly impacts outcomes, particularly after first-stage palliation, and is associated with impaired survival following Fontan completion [13,21,22]. A more differentiated examination of patient outcomes reveals that the adverse cardiovascular events vary depending on the underlying cardiac defect. While patients with HLHS more frequently suffered from cardiac decompensations and hospitalizations, resulting in more frequent resuscitations, Fontan patients with other underlying cardiac defect, such as double inlet left ventricle (DILV) or complete atrioventricular septal defect (CAVSD), more commonly experienced rhythm problems requiring pacemaker implantation and subsequent surgeries. Cardiovascular catheter interventions had the same frequency in both subgroups (approximately 30%). Patients with HLHS more often had aortic arch stenosis after aortic arch repair, while non-HLHS patients more frequently developed venovenous or aorto-pulmonary collateral vessels requiring interventional closure.

This study is limited by its retrospective nature. The numbers of patients in the subgroups were rather small. Because of this study’s retrospective nature, some patient files have missing data, and some patients were lost to follow-up from our clinic. Although the mean follow-up duration of 16 years (up to 22 years) and mean patient age of 18 years provide valuable insight into long-term Fontan outcomes, particularly for HLHS patients, the cohort remains relatively young. The higher incidence of MACE and failing Fontan may have a greater impact on morbidity and mortality with longer follow-up. Echocardiographic assessment of ventricular function in Fontan/functional single-ventricle anatomy was challenged by geometric heterogeneity, load dependence, and acoustic windows, which limit the generalizability of conventional 2D indices and contribute to measurement variability. Although we attempted to minimize subjectivity through blinded dual reading and consensus adjudication, qualitative grading remains an inherent limitation. CMR is the reference standard for ventricular volumes and function in Fontan patients, but it was not universally available at all timepoints; advanced echocardiographic techniques (3D echo, speckle-tracking strain) improved quantification when feasible, yet they were not consistently obtainable across the cohort. Additionally, diastolic ventricular function was not routinely assessed; recent studies indicate that diastolic dysfunction may emerge early in adult Fontan patients and could adversely affect outcomes [23,24]. Another limitation is the lack of adjustment for potential confounding factors such as medical therapy or comorbidities, which may have influenced long-term outcomes despite the homogeneity of perioperative management within a single center.

## 5. Conclusions

In conclusion, patients with HLHS who reach adolescence exhibit significantly higher rates of adverse cardiovascular events (MACEs), particularly cardiac decompensations with hospitalizations, which align with the findings of reduced ventricular function, higher rates of AV valve regurgitation, and elevated NT-proBNP levels.

## Figures and Tables

**Table 1:** Patient characteristics.

	Total n = 130	Dominant LV n = 72	Dominant RV (non-HLHS) n = 19	HLHS n = 39	*p*-Value
FU time (years)	16.2 ± 2.9	16.5 ± 3.0	16.8 ± 2.7	15.5 ± 2.7	0.195
Age at FU (years)	18.6 ± 3.2	19.0 ± 3.5	19.7 ± 2.9	17.5 ± 2.5	0.025
Age at TCPC	2.4 ± 1.3	2.5 ± 1.3	2.9 ± 1.6	2.0 ± 0.6	0.3
Male	88	44	12	32	0.071
Underlying cardiac anomaly					
HLHS	39			39	
TA	28	28			
DILV	18	18			
DORV	16	7	9		
CAVSD	4	2	2		
Other	25	17	8		
Other morphological characteristics					
VSD	73	51	12	10	
TAPVC	3	1	2	0	
PAPVC	2	1	1	0	
Hypoplastic aortic arch	20	10	4	6	
Heterotaxy	8	4	4	0	
Azygos continuity	7	5	2	0	

FU: follow-up, TCPC: total cavopulmonary connection, HLHS: hypoplastic left heart syndrome, TA: tricuspid atresia, DILV: double-inlet left ventricle, DORV: double-outlet right ventricle, CAVSD: complete atrioventricular septal defect, VSD: ventricular septal defect, TAPVC: total anomalous pulmonary venous connection, PAPVC: partial anomalous pulmonary venous connection.

**Table 2:** Overview of clinical findings at the latest follow-up.

	Total n = 130	Dominant LV n = 72	Dominant RV n = 19	HLHS n = 39	*p*-Value
Hospital admission after TCPC	82 (63%)	46 (64%)	13 (68%)	23 (59%)	0.275
MACE	11 (9%)	3 (4%)	2 (11%)	6 (15%)	0.039
Failing Fontan	10 (8%)	5 (7%)	1 (5%)	4 (10%)	0.489
Extended MACE	64 (49%)	36 (50%)	9 (47%)	19 (48%)	0.546
Arrhythmia	33 (35%)	18 (25%)	7 (37%)	8 (21%)	0.207
FALD	12 (9%)	4 (5%)	1 (5%)	7 (18%)	0.017
Medications					
Anticoagulation	125 (96%)	69 (96%)	17 (89%)	39 (100%)	0.774
ACE inhibitor	20 (15%)	8 (11%)	1 (5%)	11 (28%)	0.010
Diuretics	14 (11%)	8 (11%)	0	6 (15%)	0.208
Pulmonary vasodilation	10 (8%)	6 (8%)	0	4 (10%)	0.347
Beta-Blocker	20 (15%)	10 (14%)	2 (11%)	8 (21%)	0.211
Ventricular Function					
1/2/3	67/57/6	47/24/1	8/9/2	12/24/3	0.009
AVV Regurgitation					
0/1/2/3	35/61/31/3	25/33/13/1	4/11/3/1	6/17/15/1	0.044
NT-proBNP (ng/L)	248.7 (10–4140)	171.8 (10–1030)	195.9 (29–1550)	412.8 (39–4140)	0.004
Creatinine (mg/dL)	0.7 (0.3–1.9)	0.7 (0.4–1.9)	0.8 (0.5–1.1)	0.70 (0.3–1.9)	0.244
BUN (mg/dL)	30.6 (3.1–267.2)	28.5 (10.2–73.4)	28.2 (16.7–48.2)	36.1 (3.1–267.0)	0.999
AST (U/L)	33.4 (12.5–177.3)	29.9 (12.5–61.9)	29.9 (18.3–47.1)	42.1 (16.1–177.3)	0.103
ALT(U/L)	29.9 (9.5–114.0)	27.9 (9.5–53.2)	30.2 (12.8–55.9)	33.2 (11.8–114.1)	0.883
GGT(U/L)	74.8 (23.0–421.0)	62.7 (24.8–210.2)	89.0 (26.9–203.3)	90.8 (23.2–421.4)	0.120
Protein (g/dL)	7.2 (3.6–8.8)	7.2 (3.6–8.8)	7.4 (7.1–7.8)	7.1 (4.8–8.5)	0.256
Albumin (g/L)	44.8 (17.1–53.7)	44.5 (17.1–53.7)	46.6 (38.1–49.5)	44.6 (26.7–51.1)	0.435

The table shows the comparison of parameters between patients with HLHS and Fontan patients with other underlying congenital heart disease. TCPC: total cavopulmonary connection, FALD: Fontan-associated liver disease, AVV: atrioventricular valve, ventricular function 1: good, 2: slightly reduced, 3: reduced. BUN: blood urea nitrogen, ACE inhibitors: angiotensin-converting enzyme inhibitor, AST: aspartate-aminotransferase, ALT: alanine-aminotransferase, GGT: gamma-glutamyl transferase.

**Table 3:** Overview of cardiac catheter interventions in HLHS (hypoplastic left heart syndrome) and non-HLHS patients.

Cardiac Interventions	Non-HLHS (n = 91)	HLHS (n = 39)
LPA dilation/LPA stenting	9 (9.9%)	4 (10.3%)
Closure of venovenous or aorto-pulmonary collaterals	13 (14.3%)	4 (10.3%)
Aortic arch stenosis	1 (1.1%)	4 (10.3%)
TCPC tunnel ballon angioplasty/stenting	3 (3.3%)	-
Local lysis of pulmonary artery thrombus	1 (1.1%)	-
Total	27 (29.7%)	12 (30.9%)

LPA: left pulmonary artery, TCPC: total cavopulmonary connection.

## Data Availability

The data underlying this article cannot be shared publicly due to data privacy reasons and the according German regulation.

## References

[1] Fontan F., Baudet E. (1971). Surgical repair of tricuspid atresia. Thorax.

[2] Ono M., Kasnar-Samprec J., Hager A., Cleuziou J., Burri M., Langenbach C., Callegari A., Strbad M., Vogt M., Horer J. (2016). Clinical outcome following total cavopulmonary connection: A 20-year single-centre experience. Eur. J. Cardiothorac. Surg..

[3] Padalino M.A., Constantine A., Bergonzoni E., Cao I., Horer J., Ono M., Staehler H., Sames-Dolzer E., Gierlinger G., Hazekamp M. (2025). Early outcomes of children with univentricular circulation undergoing Fontan surgery: The EuroFontan registry. Eur. Heart J..

[4] Talla M., Best N., Challa A., Balakumar S., Lopez-Tejero S., Huszti E., Horlick E., Alonso-Gonzalez R., Abrahamyan L. (2025). Long-Term Outcomes of Fontan Patients with an Extracardiac Conduit: A Systematic Review and Meta-Analysis. Can. J. Cardiol..

[5] Hammer V., Schaeffer T., Staehler H., Heinisch P.P., Burri M., Piber N., Lemmer J., Hager A., Ewert P., Horer J. (2023). Protein-Losing Enteropathy and Plastic Bronchitis Following the Total Cavopulmonary Connections. World J. Pediatr. Congenit. Heart Surg..

[6] Lee T.M., Aiyagari R., Hirsch J.C., Ohye R.G., Bove E.L., Devaney E.J. (2012). Risk factor analysis for second-stage palliation of single ventricle anatomy. Ann. Thorac. Surg..

[7] Kogon B.E., Plattner C., Leong T., Simsic J., Kirshbom P.M., Kanter K.R. (2008). The bidirectional Glenn operation: A risk factor analysis for morbidity and mortality. J. Thorac. Cardiovasc. Surg..

[8] Biko D.M., DeWitt A.G., Pinto E.M., Morrison R.E., Johnstone J.A., Griffis H., O’Byrne M.L., Fogel M.A., Harris M.A., Partington S.L. (2019). MRI Evaluation of Lymphatic Abnormalities in the Neck and Thorax after Fontan Surgery: Relationship with Outcome. Radiology.

[9] Keizman E., Abarbanel I., Salem Y., Mishaly D., Serraf A.E., Pollak U. (2023). The Impact of Dominant Ventricular Morphology on the Early Postoperative Course After the Glenn Procedure. Pediatr. Cardiol..

[10] Pollak U., Abarbanel I., Salem Y., Serraf A.E., Mishaly D. (2022). Dominant Ventricular Morphology and Early Postoperative Course After the Fontan Procedure. World J. Pediatr. Congenit. Heart Surg..

[11] Ponzoni M., Azzolina D., Vedovelli L., Gregori D., Di Salvo G., D’Udekem Y., Vida V., Padalino M.A. (2022). Ventricular morphology of single-ventricle hearts has a significant impact on outcomes after Fontan palliation: A meta-analysis. Eur. J. Cardiothorac. Surg..

[12] Atz A.M., Zak V., Mahony L., Uzark K., D’Agincourt N., Goldberg D.J., Williams R.V., Breitbart R.E., Colan S.D., Burns K.M. (2017). Longitudinal Outcomes of Patients with Single Ventricle After the Fontan Procedure. J. Am. Coll. Cardiol..

[13] Abdul-Khaliq H., Gomes D., Meyer S., von Kries R., Wagenpfeil S., Pfeifer J., Poryo M. (2024). Trends of mortality rate in patients with congenital heart defects in Germany-analysis of nationwide data of the Federal Statistical Office of Germany. Clin. Res. Cardiol..

[14] Aljiffry A., Harriott A., Patel S., Scheel A., Amedi A., Evans S., Xiang Y., Harding A., Shashidharan S., Beshish A.G. (2024). Outcomes, mortality risk factors, and functional status post-Norwood: A single-center study. Int. J. Cardiol. Congenit. Heart Dis..

[15] Ghimire L.V., Chou F.S., Pundi K., Moon-Grady A.J. (2022). In-Hospital Outcomes in Fontan Completion Surgery According to Age. Am. J. Cardiol..

[16] Zoghbi W.A., Enriquez-Sarano M., Foster E., Grayburn P.A., Kraft C.D., Levine R.A., Nihoyannopoulos P., Otto C.M., Quinones M.A., Rakowski H. (2003). Recommendations for evaluation of the severity of native valvular regurgitation with two-dimensional and Doppler echocardiography. J. Am. Soc. Echocardiogr..

[17] d’Udekem Y., Iyengar A.J., Galati J.C., Forsdick V., Weintraub R.G., Wheaton G.R., Bullock A., Justo R.N., Grigg L.E., Sholler G.F. (2014). Redefining expectations of long-term survival after the Fontan procedure: Twenty-five years of follow-up from the entire population of Australia and New Zealand. Circulation.

[18] Pundi K.N., Johnson J.N., Dearani J.A., Pundi K.N., Li Z., Hinck C.A., Dahl S.H., Cannon B.C., O’Leary P.W., Driscoll D.J. (2015). 40-Year Follow-Up After the Fontan Operation: Long-Term Outcomes of 1,052 Patients. J. Am. Coll. Cardiol..

[19] Gordon-Walker T.T., Bove K., Veldtman G. (2019). Fontan-associated liver disease: A review. J. Cardiol..

[20] Wilson W.M., Valente A.M., Hickey E.J., Clift P., Burchill L., Emmanuel Y., Gibson P., Greutmann M., Grewal J., Grigg L.E. (2018). Outcomes of Patients with Hypoplastic Left Heart Syndrome Reaching Adulthood After Fontan Palliation: Multicenter Study. Circulation.

[21] Dalén M., Odermarsky M., Liuba P., Ramgren J.J., Synnergren M., Sunnegårdh J. (2024). Long-Term Survival After Single-Ventricle Palliation: A Swedish Nationwide Cohort Study. J. Am. Heart Assoc..

[22] Dib N., Chaix M.A., Samuel M., Honfo S.H., Hamilton R.M., Aboulhosn J., Broberg C.S., Cohen S., Cook S., Dore A. (2024). Cardiovascular Outcomes in Fontan Patients with Right vs Left Univentricular Morphology: A Multicenter Study. JACC Adv..

[23] Miranda W.R., Borlaug B.A., Jain C.C., Anderson J.H., Hagler D.J., Connolly H.M., Egbe A.C. (2023). Exercise-induced changes in pulmonary artery wedge pressure in adults post-Fontan versus heart failure with preserved ejection fraction and non-cardiac dyspnoea. Eur. J. Heart Fail..

[24] Hager A. (2023). Invasive Cardiopulmonary Exercise Testing in Patients with Fontan Circulation. J. Am. Coll. Cardiol..

